# Clinicopathological study of mixed cryoglobulinemic glomerulonephritis secondary to hepatitis B virus infection

**DOI:** 10.1186/s12882-020-02057-4

**Published:** 2020-09-14

**Authors:** Chao Li, Hang Li, Wei Su, Yu-bing Wen, Wei Ye, Wen-ling Ye, Jian-fang Cai, Xu-zhen Qin, Xue-mei Li, Xue-wang Li

**Affiliations:** 1 Nephrology Department, Peking Union Medical College Hospital, Chinese Academy of Medicine Sciences & Peking Union Medical College, Beijing, 100730 China; 2 Laboratory Department, Peking Union Medical College Hospital, Chinese Academy of Medicine Sciences & Peking Union Medical College, Beijing, 100730 China

**Keywords:** Cryoglobulinemia, Glomerulonephritis, Hepatitis B virus

## Abstract

**Background:**

Cryoglobulinemic glomerulonephritis (CryoGn) caused by hepatitis B virus (HBV) infection was rarely reported. Our study aimed to investigate the clinical features, renal pathology findings, and prognosis in patients with HBV related CryoGn.

**Methods:**

This was a retrospective study including seven Chinese patients with HBV related CryoGn in a tertiary referral hospital from April 2016 to March 2019. The clinical and pathological data were collected and analyzed.

**Results:**

Age at renal biopsy was 47 ± 12 years, with female/male ratio 3/4. Urine protein was 5.6 (3.0, 6.6) g/d and five cases presented with nephrotic syndrome. The baseline eGFR was 23.5 (20.2, 46.3) ml/min per 1.73m^2^. The extrarenal manifestations included purpura (*n* = 6), arthralgia (*n* = 1), peripheral neuropathy (*n* = 1), and cardiomyopathy (*n* = 1). Six cases had type II cryoglobulinemia with IgMκ, the other one had type III. The median cryocrit was 4.0 (1.0, 15.0) %. Renal pathologic findings on light microscopy: endocapillary proliferative glomerulonephritis (Gn) (*n* = 3), membranoproliferative Gn (*n* = 3), and mesangial proliferative Gn (*n* = 1). On immunofluorescence microscopy, the predominant type of immunoglobulin deposits was IgM (*n* = 5). HBsAg and HBcAg deposits were found in one case. Ultrastructural studies showed granular subendothelial and mesangial electron-dense deposits in all patients and microtubules in one case. All patients received antiviral medications. They were given corticosteroid alone (*n* = 2) or combined with cyclophosphamide (*n* = 4) or mycophenolate mofetil (*n* = 1). Two patients received plasmapheresis. The median follow-up time was 18 (6, 37) months. Four patients got remission, two patients died of pneumonia, and one progressed to end-stage renal disease (ESRD). At endpoint of follow-up, 24hUP was 2.1 (0.8–5.2) g/d, and eGFR was 55.3 (20.7, 111.8) ml/min per 1.73m^2^. The median cryocrit decreased to 1.0 (0, 5.75) %.

**Conclusions:**

The etiology of mixed CryoGn should be screened for HBV infection. Endocapillary proliferative Gn and membranoproliferative Gn were the common pathologic patterns. Diagnosis and treatment in early stage benefit patients’ renal outcomes. Immunosuppressive therapy should be considered for severe renal disease, based on efficient antiviral therapy.

## Background

Glomerulonephritis (Gn) is one of the extrahepatic complications associated with the hepatitis B virus (HBV). Several morphologic patterns of glomerulonephritis associated with HBV infection have been reported since 1971. Membranous nephropathy was the most common morphological pattern of renal disease in HBV, while cryoglobulinemic Gn (CryoGn) associated HBV infection was also described in some case reports [1, 2]. Although a relatively high prevalence of HBV infection in Asia, CryoGn associated with chronic HBV infection has been rarely reported systematically in the literature. The natural history, optimal therapy of HBV related CryoGn are also unclear.

The objective of our study is to investigate clinical and histological features, treatment and outcome of CryoGn associated with chronic HBV infection in a single medical center in China.

## Methods

### Study population

We performed a retrospective study on patients with biopsy-proven CryoGn at Peking Union Medical College Hospital, a tertiary referral hospital, between April 2016 and March 2019. Inclusion criteria were as follows: 1) cryoglobulin was positive. 2) evidence of chronic hepatitis B infection. Other secondary causes of mixed cryoglobulinemia (MC) were excluded. HCV-antibody was routinely done to exclude HCV. Anti-nuclear antibody (ANA), anti-double-stranded deoxyribonucleic acid (dsDNA), and anti-extractable nuclear antigen (ENA) antibody profiles were examined to exclude Systemic Lupus Erythematosus (SLE) and Sjögren’s syndrome. For screening lymphoproliferative neoplasms, we checked blood smear, and bone marrow smear in the presence of monoclonal gammopathy. If lymphoma was suspected, ultrasound and computed tomography (CT) were commonly used as a guide for a diagnostic biopsy and positron emission tomography (PET)-CT sometimes was implemented.

The renal biopsy specimens of all the patients were examined by light microscopy, immunofluorescence and electron microscopy. Two renal pathologists reviewed the renal biopsy specimens independently.

Clinical and laboratory data of included patients were recorded at the time of the initial evaluation, during follow-up and at the end of follow-up (last visit, or date of death). The following data were collected: gender, age at diagnosis, dates of renal biopsy, blood pressure, cutaneous involvement (purpura, distal ulcers, necrosis), neurologic involvement (polyneuropathy, multiple mononeuritis and central nervous system involvement), renal involvement (proteinuria, hematuria, elevated serum creatinine level), musculoskeletal involvement (arthralgia, arthritis, myalgia), gastrointestinal involvement, and outcome. Laboratory evaluation included serum C3 and C4, rheumatoid factor (RF), serum immunoglobulin levels (IgG, IgA and IgM), cryoglobulin, serum HBV antigens and antibodies profiles (HBsAg, HBeAg, HBsAb, HBcAb, HBeAb), serum HBV DNA quantification, serum creatinine (Scr), serum albumin, and 24-h urine protein (24hUP). The estimated glomerular filtration rate (eGFR) was calculated by Chronic Kidney Disease-Epidemiology Collaboration (CKD-EPI) equation.

The regimen choice was decided by renal physicians with consideration of the patient’s preference and drug potential side effect. The included patients were followed up regularly. The last follow-up date of our study was on Dec 31st, 2019.

### Renal pathology

Tissue for light microscopy was routinely fixed in formalin and embedded in paraffin. The paraffin sections were cut at 2 μm and stained with hematoxylin and eosin (H&E), periodic acid-Schiff (PAS), periodic acid-silver methenamine (PASM) and Masson’s trichrome stains.

Specimen for immunofluorescence (IF) was cut at 5 μm in freezing microtome and stained with fluorescein isothiocyanate (FITC) -conjugated antibodies to IgG, IgA, IgM, C3, C4, C1q, κ- and λ-light chains, fibrinogen and albumin (Dako), as well as to HBsAg and HBcAg (Abcam).

Renal tissues for electron microscopy were performed according to standard protocol using Spurr’s resin for embedding, osmium tetroxide post-fixation and uranyl acetate contrasting.

### Cryoglobulins measurement

Serum cryoglobulins were measured according to standard techniques reported in the literature [3]. Blood samples were collected and transferred at 37 °C. After clotting and centrifugation at 37 °C, serum was incubated at 4 °C for 1 week. The amount of cryoglobulin is measured by the percentage level of cryoprecipitate in the Wintrobe tube. After isolation and washing, immunotyping of the cryoprecipitate was analyzed by capillary electrophoresis (CAPILLARYS 2 FLEX PIERCING, Sebia, France).

### Definition of variables

MC was classified according to the Brouet classification [4]. Types II is characterized by polyclonal IgG and monoclonal IgM. Type III is immunocomplexes composed of polyclonal IgG and polyclonal IgM.

Complete remission (CR) was defined as a reduction in urine protein excretion to < 0.5 g/day together with improved or stable eGFR. Partial remission (PR) was defined as a reduction > 50% of the baseline proteinuria (with a lower value of < 3.5 g/d) with stable eGFR. A stable eGFR was defined as a eGFR remaining unchanged or declines by < 15% during follow-up.

### Statistical analyses

Baseline data and outcome were expressed as percentages for categorical variables, means and standard deviations (SD) for normally distributed continuous variables, or median and quartiles (P25, P75) for non-normally distributed continuous variables. All statistical analyses were performed by SPSS 22.0 (IBM Corporation, NY, USA), and *P*-values of 0.05 (two-tailed) were considered statistically significant.

## Results

### Patients and clinical characteristics

Our study included seven patients (three females and four males) with a median age of 45 years old. Tables 1 and 2 showed patients’ demographic, clinical characteristics and laboratory data. Five cases presented with nephrotic syndrome and the other two presented with nephritic and RPGN, respectively. As for extrarenal involvement, six patients presented purpura. Peripheral neuropathy was found in one case, as well as cardiomyopathy in another one. All patients were HBsAg seropositive and two of them were HBeAg seropositive. HBV-DNA test showed virus active replication in three patients, although their transaminase levels were normal without cirrhosis evidence in ultrasound. Six patients had definite chronic hepatitis B infection history with a median 20 (9.5, 24.8) years, while HBV infection history of the remaining one patient was unknown.

**Table 1:** Clinical data of seven patients with mixed cryoglobulinemic glomerulonephritis associated with HBV infection

Case	Gender	Age (yrs)	Kidney disease history before renal biopsy (months)	Clinical renal syndrome	HTN	Extrarenal involvement	HBsAg	HBsAb	HBeAg	HBeAb	HBcAb	HBV-DNA (copies/ml)
1	Female	40’s-50’s	60	NS	Yes	Cutaneous, Articular	+	–	–	+	+	< 10^3^
2	Female	50’s-60’s	0.5	RPGN	Yes	Cutaneous, Peripheral nerve	+	–	–	+	+	< 10^3^
3	Female	30’s-40’s	1	NS	Yes	None	+	–	+	–	+	1.14 × 10^6^
4	Male	60’s-70’s	4	NS	Yes	Cutaneous	+	–	–	+	+	< 10^3^
5	Male	20’s-30’s	43	NS	No	Cutaneous, Cardiac	+	–	+	–	+	5 × 10^6^
6	Male	50’s-60’s	36	Nephritic	Yes	Cutaneous	+	–	–	+	+	< 10^3^
7	Male	40’s-50’s	240	NS	Yes	Cutaneous	+	–	–	+	+	2.27 × 10^8^

*NS* Nephrotic syndrome, *RPGN* Rapidly progressive glomerulonephritis, *HTN* Hypertension

**Table 2:** Laboratory features of seven patients with mixed cryoglobulinemic glomerulonephritis associated with HBV infection

Case	Hb(g/L)	Scr (μmol/L)	eGFR (ml/min per 1.73m^**2**^)	SAlb(g/L)	24hUP(g/d)	C3(g/L)	C4(g/L)	RF (IU/ml)	Cryoglobulin typing	Cryocrit(%)	M protein in SPE(g/L)	SIFE	UIFE	Serum IgM(g/L)
1	84	111	51.07	34	3.29	0.519	0.005	361	II, IgMκ	5	0.20	–	F-κ	0.80
2	91	320	13.14	28	3.03	0.315	0.003	564	II, IgMκ	23	–	–	–	2.32
3	100	223	23.50	33	0.43	0.405	0.001	3752	II, IgMκ	15	7.20	IgMκ	F-κ	15.36
4	82	151	40.59	24	11.57	0.566	0.035	733	II, IgMκ	4	3.60	IgMκ	–	4.74
5	99	169	46.26	25	5.57	0.444	0.013	368	II, IgMκ	4	0.40	IgMκ	–	4.53
6	83	284	21.00	24	6.57	0.480	0.138	103	II, IgMκ	1	–	–	–	0.82
7	99	306	20.20	30	6.19	0.726	0.118	117	III	1	–	–	–	1.40

*Hb* Hemoglobulin, *Scr* Serum creatinine, *eGFR* Estimated glomerular filtration rate, *SAlb* Serum albumin, *24hUP* 24-h urine protein, *RF* Rheumatoid factor, *SIFE* Serum immunofixation electrophoresis, *UIFE* Urine immunofixation electrophoresis

Anemia occurred in all patients. The median baseline eGFR (CKD-EPI) was 23.5(20.2, 46.3) ml/min per 1.73m^2^. 24hUP was 5.6(3.0, 6.6) g/d. Six cases (86%) had type II MC (IgMκ) and the other one (14%) had type III MC. Serum complement C4 levels were extremely low (median level 0.013 g/L) in all cases. Rheumatoid factors were present with high levels (median 368 IU/ml) in all patients. Serum IgM elevated in four patients.

### Renal histopathological features

The renal pathological features of seven patients were summarized in Table 3. Pathological patterns on light microscopy included Membranoproliferative glomerulonephritis (*n* = 3), endocapillary proliferative glomerulonephritis (*n* = 3) and mesangial proliferative glomerulonephritis (*n* = 1). The capillary lumen showed PAS-positive hyaline thrombi in four patients. Crescents were seen in two patients.

**Table 3:** Renal pathology findings of seven patients with mixed cryoglobulinemic glomerulonephritis caused by HBV

Case	Immunofluorescence								Light microscopy			Electron microscopy
	IgG	IgA	IgM	C3	C4	C1q	HBsAg	HBcAg	Type	Hyaline thrombi	Crescent	
1	–	1 + ~ 2+	–	1 + ~ 2+	–	–	–	–	MPGN	+	–	DD
2	2+	2 + ~ 3+	4+	1+	±	2+	1+	1 + ~ 2+	EPGN	+	–	microtubule
3	1+	±	1+	1 + ~ 2+	–	–	–	–	EPGN	+	–	DD
4	1+	–	3+	2+	–	–	–	–	MPGN	+	+	DD
5	–	–	1+	± ~ 1+	–	1+	–	–	EPGN	–	–	DD
6	1+	1+	–	±	±	±	–	–	MPGN	–	+	DD
7	1+	–	2+	–	–	–	–	–	MesPGN	–	–	DD

*MPGN* Membranoproliferative glomerulonephritis, *EPGN* Endocapillary proliferative glomerulonephritis, *MesPGN* Mesangial proliferative glomerulonephritis, *DD* Dense deposit

Immunofluorescence demonstrated subendothelial and intracapillary glomerular deposits of IgG, IgA, IgM, C3 or C4. However, we only observed glomerular HBsAg and HBcAg deposition in one patient.

Electron microscopy showed granular electron-dense deposits in glomeruli in all patients. We also found the subendothelial microtubule structure in one patient.

### Treatment and clinical outcomes

**Fig. 1:**
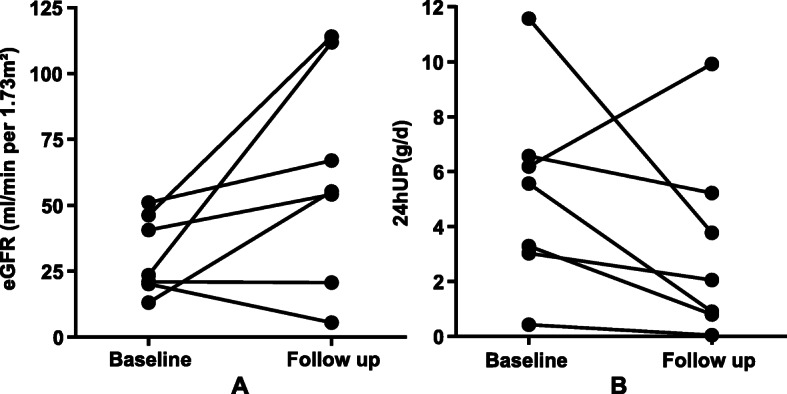
The change of eGFR (**a**) and proteinuria (**b**) in seven mixed cryoglobulinemic glomerulonephritis secondary to HBV infection at baseline and endpoint of follow-up. eGFR: estimated glomerular filtration rate. 24hUP: 24-h urine protein

Treatment and follow-up data of seven patients were summarized in Table 4. All patients received antiviral medication (entecavir, *n* = 6; lamivudine, *n* = 1). For the patients with relatively stable serum creatinine, the antiviral medication was given at least 2 weeks earlier before starting immunosuppressive therapy to evaluate whether HBV viral suppression could induce remission of kidney injury. And for those presenting with rapidly progressive glomerulonephritis, severe nephrotic syndrome or along with other severe extra-renal involvement, we initiated immunosuppressive therapy concomitantly with antiviral therapy. They were given corticosteroid alone (*n* = 2) or combined with cyclophosphamide (*n* = 4) or mycophenolate mofetil (*n* = 1). Besides, two patients received plasmapheresis for six and eight sessions respectively during hospitalization. The median follow-up time was 18 (6, 37) months. Two patients died from pneumonia, and one patient progressed to end-stage renal disease (ESRD). We repeated HBV-DNA and HBsAg in five survival patients during follow-up. HBV-DNA levels were undetectable after antiviral treatment in four patients. However, HBsAg seroconversion was not observed in any patient. Figure 1 showed the trend of proteinuria and eGFR before and after immunosuppressive therapy. At endpoint of follow-up, 24hUP was 2.1 (0.8–5.2) g/d, and eGFR (CKD-EPI) was 55.3 (20.7, 111.8) ml/min per 1.73m^2^. The median cryocrit decreased to 1.0 (0, 5.75) %.

**Table 4:** Treatment and outcome of seven patients with mixed cryoglobulinemic glomerulonephritis associated with HBV infection

Case	Anti-viral	Immunosuppressive therapy	Follow-up time (months)	Scr (μmol/L)	eGFR (ml/min per 1.73m^**2**^)	24hUP (g/d)	Cryocrit(%)	HBV-DNA (copies/ml)	Outcome
1	ETV	CS + MMF	37	90	67.00	0.80	NA	< 10^3^	PR
2	ETV	CS + CYC + DFPP	22	99	55.30	2.05	7.0	< 10^3^	PR
3	ETV	CS + CYC + DFPP	6	60	111.80	0.05	0	< 10^3^	CR
4	LAM	CS + CYC	3	119	54.13	3.77	NA	NA	death
5	ETV	CS	62	80	114.00	0.90	2.0	1.24 × 10^4^	PR
6	ETV	CS	12	287	20.70	5.22	NA	NA	death
7	ETV	CS + CYC	18	900	5.50	9.92	0	< 10^3^	dialysis

*Scr* Serum creatinine, *eGFR* Estimated glomerular filtration rate, *24hUP* 24-h urine protein, *ETV* Entecavir, *LAM* Lamivudine, *CS* Corticosteroid, *MMF* Mycophenolate, *CYC* Cyclophosphamide, *DFPP* Double-filtration plasmapheresis, *CR* Complete remission, *PR* Partial remission, *NA* Not available

## Discussion

In 1977, Y. Levo and his coworkers firstly described the association between HBV infection and MC [1]. It has been established that HBV is a rare infectious etiology of MC, compared with HCV infection. It was unknown what percentage of patients with chronic HBV infection may develop CV. A search of the literature revealed few studies focusing on CryoGn associated with HBV infection. Our retrospective study described the spectrum of clinical presentations and pathological features of seven Chinese patients with HBV related CryoGn, as well as their response to therapy and renal outcome.

We found that CryoGn associated with HBV were commonly associated with type II cryoglobulins. This finding was consistent with that of Italian study which reported type II cryoglobulins accounted for 88% in HBV related cryoglobulinemic vasculitis [5]. However, result in another previous Chines study suggested that type III cryoglobulinemia was more frequently seen in HBV related CryoGn [6]. This discrepancy may reflect differences in the laboratory methods in detection of cryoglobulin as well as patient selection bias considering a limited sample size. The most common extrarenal manifestation was cutaneous lesions (6/7) in our study, while other organs were seldom involved. Considering that only one case with Type III MC was included in our study, we were not able to conclude that clinical manifestation of the vasculitis is more frequently in Type II MC than in Type III MC reported in the literature [7].

Nephrotic syndrome was the most common syndrome with microscopic hematuria in our included patients. Acute renal injury or chronic renal insufficiency also occurred in most patients and some of them presented as RPGN and needed renal replacement therapy. We found HBsAg in the cryoprecipitate in two of our patients, confirming the pathogenic role of HBV in cryoglobulinemia.

CryoGn with a membranoproliferative pattern of injury was mostly reported in the literature, no matter what etiology it is [8, 9]. Our data demonstrated that endocapillary proliferative Gn was also the common morphologic type on light microscopy, as well as membranoproliferative Gn. The morphologic pattern of HBV related CryoGn varied in the limited number of previous studies. Membranous nephropathy was the predominant pattern reported in one study including eight Chinese cases of CryoGn associated with HBV infection [10], while the result of another Chinese study demonstrated that membranoproliferative GN was the exclusive type of all 12 patients [6]. The discrepancy of the morphologic pattern of CryoGn among different studies could have been generated by limited sample size. Hyaline thrombi were characteristic findings of CryoGn on light microscopy but inconsistently occurred in our study. And we didn’t find the association between the nephritic syndrome and the presence of hyaline thrombi.

On immunofluorescence microscopy, mesangial and capillary wall deposits of immunoglobulins (IgM, IgG or IgA) and C3 were found in most of the patients, which indicated the subendothelial location of deposits. However, it was notable that HBsAg and HBcAg staining were positive only in one patient in our study, which was similar to the results of a previous study [10]. We don’t find the specific reason for the absence of HBV antigen deposition in glomeruli in most patients with CryoGn associated with HBV infection. It is possible that circulating immune complexes of HBV antigen and antibodies plays a less important role than cryoglobulins in the pathogenesis of CryoGn. Another possible explanation for these results may be that cryoglobulins interfere combination between antigen and antibody, which decreased the sensitivity of immunofluorescence staining of HBsAg and HBcAg.

Diagnostic features for CryoGn by electron microscopy include microtubular, fibrillary, and crystal structure, but these structures are not always observed [8]. Moreover, these features are not specific to the etiology of CryoGn. All the included patients in our study had granular electron-dense deposits located in the subendothelial region. We observed microtubule structure in only one case on electron microscopy. These findings were consistent with the ultrastructural features of HBV related CryoGn reported in the literature [6, 10]. In one previous study including 12 patients of CryoGn associated with HBV infection, only two cases were found microtubule substructure on electron microscopy [6].

The optimal treatment for HBV-associated CryoGn is not known since there is no randomized trial. This is probably due to the uncommon occurrence of HBV-associated cryoglobulinemic vasculitis. Several studies confirmed a correlation between HBV suppression and CV regression after nucleoside analogs (NAs) therapy [5, 11, 12]. These findings support the hypothesis that HBV replication plays an important role in the CV etiopathogenesis. Among different NAs, entecavir is preferred given its antiviral efficacy, low propensity for drug resistance, as well as low risk for nephrotoxicity [13]. Considering limited data and potential side effects, interferon-alfa is not recommended for the treatment of HBV-associated CryoGn [5]. In our study, most of the patients (6/7) received entecavir. And three patients with elevated HBV-DNA at baseline were well controlled and HBV-DNA was undetectable at the endpoint of our study.

The studies in the literature demonstrated that NAs therapy in HBV-related CV yields high virologic and satisfying clinical responses in most patients with mild and moderate CV, but a low response in the severe CV. For patients with severe disease, immunosuppressive therapy is recommended, based on efficient antiviral therapy to prevent HBV reactivation [14]. Immunosuppressive therapy targets the inflammatory cells involving in the pathogenesis of vasculitis. In our study, five patients’ eGFR improved and four of them achieved complete or partial remission after receiving immunosuppressive therapy. Their renal outcomes were favorable during follow-up. Notably, using corticosteroids without NAs is associated with refractory or relapsing disease. The Number Seven case in our study initially received corticosteroid and cyclophosphamide without antiviral therapy because HBV serologic tests were neglected in a local hospital which led to HBV reactivation and kidney disease relapse. Although we added entecavir to his treatment in our clinic as soon as his HBV serological result was available, he had a poor response to therapy and progressed to ESRD during follow-up. Although one case report described the efficacy and safety of rituximab in HBV-related CryoGn [15], no patients in our study were received this biologic considering the potential risk of HBV reactivation. Moreover, the side effect of conventional immunosuppressive therapy, such as severe infection, should be considered before initiation especially in elderly patients with renal insufficiency. During follow-up, two patients died of pneumonia although there was no definite evidence of bone marrow suppression.

Two patients in our study received plasmapheresis to remove circulating cryoglobulins. Notably, we usually use the heating unit and warmed replacement fluid to prevent the precipitation of cryoglobulin in the pipeline. Although we didn’t find the significant association between eGFR level and the concentration of cryoglobulins, their eGFR improved accompanied by cryoglobulin level decline after six or eight sessions of plasmapheresis during hospitalization. Limited data in the literature suggested clinical improvement in CV patients with severe organ involvements after plasmapheresis [16–18]. A multicenter retrospective study in Italy recommended plasmapheresis in patients with severe CV, especially in cases with acute kidney injury, to prevent irreversible lesions [19]. Considering that plasmapheresis doesn’t inhibit the generation of new cryoglobulins, it is rational to combine with other immunosuppressive therapy to reduce the production of cryoglobulins.

The major limitations of our study included the small sample size and relatively short follow-up time. All patients in our study had normal liver function without cirrhosis evidence. Therefore, caution must be applied, as the findings and immunosuppressive therapy in our study might not be transferable to HBV patients of CryoGn with evidence of active hepatitis or severe cirrhosis. Further prospective studies are needed to evaluate the efficacy and safety of corticosteroid or immunosuppressors in patients with HBV related CryoGn.

## Conclusions

The etiology of mixed CryoGn should be screened for HBV infection. Endocapillary proliferative Gn and membranoproliferative Gn were the common pathologic patterns. Diagnosis and treatment in early stage benefit patients’ renal outcomes. Immunosuppressive therapy should be considered for severe renal disease, based on efficient antiviral therapy.

## Data Availability

Comprehensive data presented in manuscript, tables and figures. Additional data available on request from the corresponding author.

## Abbreviations

CryoGn: Cryoglobulinemic glomerulonephritis
MC: Mixed cryoglobulinemia
HBV: Hepatitis B virus
HCV: Hepatitis C virus
ANA: Anti-nuclear antibody
dsDNA: Double-stranded deoxyribonucleic acid
ENA: Extractable nuclear antigen
SLE: Systemic Lupus Erythematosus
CT: Computed tomography
PET: Positron emission tomography
24hUP: 24-h urine protein
ESRD: End-stage renal disease
Gn: Glomerulonephritis
Scr: Serum creatinine
eGFR: Estimated glomerular filtration rate
CKD-EPI: Chronic Kidney Disease-Epidemiology Collaboration
SIFE: Serum immunofixation electrophoresis
UFIE: Urine immunofixation electrophoresis
H&E: Hematoxylin and eosin
PAS: Periodic acid-Schiff
PASM: Periodic acid-silver methenamine
IF: Immunofluorescence
FITC: Fluorescein isothiocyanate
CR: Complete remission
PR: Partial remission
SD: Standard deviation
NS: Nephrotic syndrome
RPGN: Rapidly progressive glomerulonephritis
MPGN: Membranoproliferative glomerulonephritis
EPGN: Endocapillary proliferative glomerulonephritis
MesPGN: Mesangial proliferative glomerulonephritis
DD: Dense deposit
ETV: Entecavir
LAM: Lamivudine
CS: Corticosteroid
MMF: Mycophenolate
CYC: Cyclophosphamide
DFPP: Double-filtration plasmapheresis
